# Patterns of Bullying Victimization and Associations with Mental Health Problems in Chinese Adolescents: A Latent Class Analysis

**DOI:** 10.3390/ijerph17030779

**Published:** 2020-01-27

**Authors:** Sheng Zhang, Meiqian Gong, Wenyan Li, Wanxin Wang, Ruipeng Wu, Lan Guo, Ciyong Lu

**Affiliations:** Department of Medical statistics and Epidemiology, School of Public Health, Sun Yat-sen University, Guangzhou 510080, China; zhangsh46@mail2.sysu.edu.cn (S.Z.); gongmq@mail2.sysu.edu.cn (M.G.); liwy23@mail2.sysu.edu.cn (W.L.); wgg0808@163.com (W.W.); wurp5@mail2.sysu.edu.cn (R.W.); guolan3@mail.sysu.edu.cn (L.G.)

**Keywords:** bullying victimization, latent class analysis, mental health problems, adolescents

## Abstract

Bullying victimization in school students is a serious public health concern and has been linked to a wide range of mental health problems. The current study aims to examine patterns of involvement in different types of bullying victimization among Chinese adolescents and evaluate the associations between bullying victimization and mental health problems. Cross-sectional data from 20,722 middle school students from Guangdong Province were sampled using a multistage, stratified cluster-randomized sampling method. Latent class analysis (LCA) was performed on seven items representing bullying victimization. Levels of mental health outcomes were compared across each latent class. Four latent classes were identified for boys: the high victimization class (0.6%), the moderate victimization class (2.8%), the verbal victimization class (12.4%), and the low victimization class (84.2%). For girls, three latent classes were identified: the high victimization class (0.7%), the moderate victimization class (5.6%), and the low victimization class (93.7%). Characteristics of the item probabilities were different between boys and girls. For both genders, a graded relationship was found between bullying victimization class membership and mental health outcomes. These findings underline the complexity of bullying victimization patterns among Chinese adolescents. Students with higher involvement in bullying victimization have more severe mental health problems.

## 1. Introduction

Bullying victimization constitutes a serious public health concern in school students around the world. According to Olweus, bullying victimization is defined as the experience of intentional, repetitive, and aggressive actions by peers towards an individual who has difficulty defending himself or herself [1]. A previous study found adolescents to be the most common targets of bullying behaviors [2]. It was reported that approximately 32% of students across 38 countries had experienced being bullied [3]. In China, the issue also seems to be prevalent, as approximately half of students have reported lifetime bullying victimization [4,5].

Several types of bullying behaviors have been identified, such as physical, verbal, relational, and cyber behaviors. Traditional types of bullying include physical (hitting, kicking, pushing, etc.), verbal (name-calling, taunting, etc.), and relational (spreading rumors, excluding, etc.) bullying [6]. Physical and verbal aggression are direct forms of bullying that involve face-to-face confrontation, whereas relational aggression is regarded as indirect bullying [7]. Cyber bullying refers to an emerging form of bullying through the application of computers, cell phones, and other electronic devices [8].

The literature has suggested that gender differences exist in the occurrence of different types of bullying victimization. Olweus reported that, in Norway, boys were more likely to be victims of physical and verbal bullying, whereas girls were more vulnerable to relational bullying [9]. In a survey of Finnish children, boys reported more physical victimization and girls reported more relational victimization, but there was no significant difference in verbal victimization [10]. The same results have been found in British studies [11,12].

The impact of bullying victimization on children and adolescents has been linked to a wide range of negative mental health outcomes. Studies have found that victims of bullying are particularly vulnerable to serious mental health problems such as anxiety and depression [13,14]. More seriously, bullying victimization is a significant risk factor for non-suicidal self-injury (NSSI), suicidal ideation and behaviors [15,16,17]. Physiological responses to stress, cognitive distortion, and oversensitivity to social cues may be potential mechanisms for the associations between bullying victimization and mental health problems [18]. As mental disorders in children and adolescents have been increasing over recent decades [19], bullying prevention programs may be important in promoting mental health of victims of bullying [20].

Considering that some individuals may be the victims of multiple types of bullying while others may be the victims of only one type, some recent studies have explored the application of latent class analysis (LCA) to better understand the co-occurrence of different types of bullying victimization [21,22]. Similar to cluster analysis, LCA is a person-centered analysis approach that aims to classify samples into several mutually exclusive classes depending on their observed responses to multiple manifest indicators [23]. In an LCA model, a latent categorical variable is created to determine individuals’ class membership, and their associations with predictor variables or outcome variables can be tested. This approach has been validated in numerous studies of health-related behaviors [24,25,26,27]. LCA has been conducted in previous studies to examine patterns of bullying victimization in US adolescents [21,22]. However, patterns of bullying victimization among Chinese youth and the associations of bullying victimization with mental health problems remain unclear. Therefore, we conducted this large-scale survey and employed LCA to examine patterns of involvement in different types of bullying victimization among Chinese adolescents and to evaluate the associations between bullying victimization and an array of mental health outcomes including anxiety symptoms, depressive symptoms, NSSI, suicidal ideation, and suicide attempts.

## 2. Materials and Methods

### 2.1. Study Design and Participants

The data were collected from a school-based adolescent health survey in Guangdong Province in 2018. Participants were selected through a multistage, stratified cluster-randomized sampling method. In stage 1, the entire province was divided into three economic stratifications by per capita GDP (high, medium, and low) and then two cities were randomly selected from each stratification. In stage 2, schools in each selected city were divided into three categories: junior high schools, senior high schools, and vocational high schools. We randomly selected six junior high schools, four senior high schools, and two vocational high schools based on the overall proportions of the three types of schools. In stage 3, two classes were randomly selected from each grade of the selected schools and all available students in the selected classes were invited to voluntarily participate in this study. Among the 21,982 students who were invited to participate, 20,722 students completed the questionnaires and qualified for our study, resulting in a response rate of 94.3%.

The anonymous self-report questionnaires were completed during one class period (45 min). Research assistants administered the survey in the classroom without the presence of teachers to avoid any potential information bias. The study protocol was approved by the Institutional Review Board of Sun Yat-Sen University, School of Public Health. Written informed consent was obtained from each student and one of his or her parents prior to the survey.

### 2.2. Measures

#### 2.2.1. Bullying Victimization

Bullying victimization was measured by seven victimization items adapted from the Chinese version of the Olweus Bully/Victim Questionnaire (OBVQ) [28], which has been applied among Chinese adolescents in previous studies with good internal consistency and test-retest reliability [29,30]. For each item, students were asked how often they were bullied in a specific way during the current semester. The seven items were as follows: item 1—“I was called mean names, was made fun of, or teased in a hurtful way”; item 2—“Other students left me out of things on purpose, excluded me from their group of friends, or completely ignored me”; item 3—“I was hit, kicked, pushed, shoved around, or threatened”; item 4—“Other students told lies or spread false rumors about me and tried to make others dislike me”; item 5—“I had money or other things taken away from me or damaged”; item 6—“I was bullied with mean names or comments about my accent”; and item 7—“I experienced cyberbullying (being bullied via text message, E-mail, a social network or a website)”. Verbal victimization was measured by item 1 and item 6, physical victimization was measured by item 3 and item 5, relational victimization was measured by item 2 and item 4, and cyber victimization was measured by item 7. The response options were “never”, “only once or twice”, “two or three times a month”, “about once a week”, and “several times a week”. Each item was converted to a dichotomous variable (0 = uninvolved, 1 = involved) based on the cutoff point of the frequency of “two or three times a month” [28] and was included in the latent class models as a categorical manifest indicator.

#### 2.2.2. Anxiety Symptoms

Students’ anxiety symptoms were assessed using the Chinese version of the 7-item Generalized Anxiety Disorder scale (GAD-7) [31]. The GAD-7 reflects how often the subjects have suffered from the seven core symptoms of generalized anxiety disorder over the past two weeks. The response options include “not at all”, “several days”, “more than half the days”, and “nearly every day”, which are scored as 0, 1, 2, and 3, respectively. GAD-7 scores range from 0 to 21 with higher scores representing more severe anxiety symptoms.

#### 2.2.3. Depressive Symptoms

The Chinese version of the Center for Epidemiology Scale for Depression (CES-D) [32] was utilized to evaluate the level of the students’ depressive symptoms. The respondents were asked to rate the frequency of occurrence of 16 negative items and four positive items over the past week by choosing one of the following response options: “rarely or none of the time (less than 1 day)”, “some or a little of the time (1–2 days)”, “occasionally or a moderate amount of the time (3–4 days)”, or “most or all of the time (5–7 days)”. The total scores range from 0 to 60 and higher scores indicate more severe depressive symptomatology.

#### 2.2.4. NSSI, Suicidal Ideation, and Suicide Attempts

NSSI was assessed by the question, “During the past 12 months, how many times did you do something to purposely hurt yourself without wanting to die, such as cutting or burning yourself on purpose?” Suicidal ideation was assessed by the question “During the past 12 months, did you ever seriously consider attempting suicide?” Suicide attempts were assessed by the question, “During the past 12 months, how many times did you actually attempt suicide?” Responses to the above three questions were all dichotomized as “yes” or “no”.

### 2.3. Statistical Analysis

LCA was employed to identify the underlying homogenous subgroups of students based on their responses to the seven bullying victimization items and to examine the relationships between bullying victimization class membership and mental health outcomes. To explore whether there were gender differences in patterns of bullying victimization, LCA was performed separately by gender. The most appropriate number of latent classes was determined through comparisons of several model fit indices, including the Akaike information criterion (AIC), Bayesian information criterion (BIC), sample size adjusted Bayesian information criterion (SSABIC), entropy, Lo-Mendell-Rubin adjusted likelihood ratio test (LMR), and bootstrapped likelihood ratio test (BLRT) [33]. Lower AIC, BIC, and SSABIC values suggest better model fit. The entropy value of each model ranges from 0 to 1, with a higher value indicating better classification accuracy. The LMR and BLRT were used to compare the k-class model with the (k-1)-class model, and non-significant values for the k-class model indicate that the model with (k-1) classes should be more parsimonious. After the number and nature of victimization classes were determined, individuals were assigned to a specific class according to their highest posterior probabilities. Next, we tested the association between class membership and levels of mental health outcomes. For continuous outcome variables, including the GAD-7 scores and CES-D scores, the mean differences across classes were tested using the modified BCH method [34]. For binary outcome variables, including NSSI, suicidal ideation and suicide attempts, the probabilities were compared across classes using Lanza’s method [35]. Data analyses were conducted using Mplus version 7.4 (Muthén and Muthén).

## 3. Results

### 3.1. Descriptive Characteristics

The characteristics of the sample are shown in Table 1. Of the total sample included in the analyses, 50.3% were males and 49.7% were females. The mean age of the participants was 15.0 years old, with an SD of 1.79. The involvement rates for the seven victimization items ranged from 1.1% to 11.2%, and the two most commonly endorsed items were a verbal victimization item (item 1) and a relational victimization item (item 4). The mean (SD) GAD-7 scores and CES-D scores were 4.15 (4.4) and 14.36 (10.2), respectively. A total of 13.9%, 17.8% and 3.6% of the students reported histories of NSSI, suicidal ideation, and suicide attempts during the past 12 months, respectively. Significant gender differences were found for all variables (*p* < 0.05). Boys had a higher involvement rate for each victimization item, whereas mental health problems were more prevalent among girls.

### 3.2. Latent Class Analysis

The fit indices for the series of latent class models are presented in Table 2. All fit indices were taken into consideration but the SSABIC value was emphasized according to the recommendation of a previous study [36], and the four-class solution for boys and the three-class solution for girls were considered to be the best models. For boys, the four-class model had the lowest SSABIC value, suggesting the best fit to the data. Additionally, the LMR and BLRT values were no longer significant for the five-class model, which indicated that the four-class model was the most parsimonious. For girls, the SSABIC value of the three-class model was the lowest, and the LMR value approached non-significance (*p* = 0.049) for the four-class model, providing support for the selection of the three-class model.

**Figure 1:**
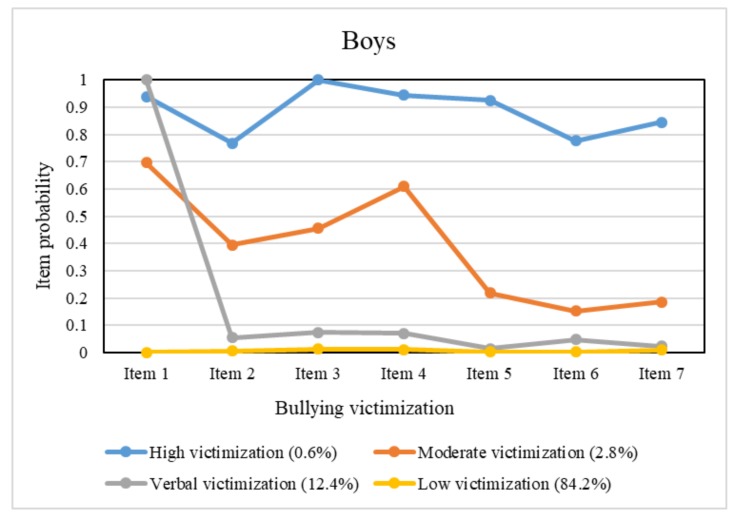
Conditional item probability plots for boys.

**Figure 2:**
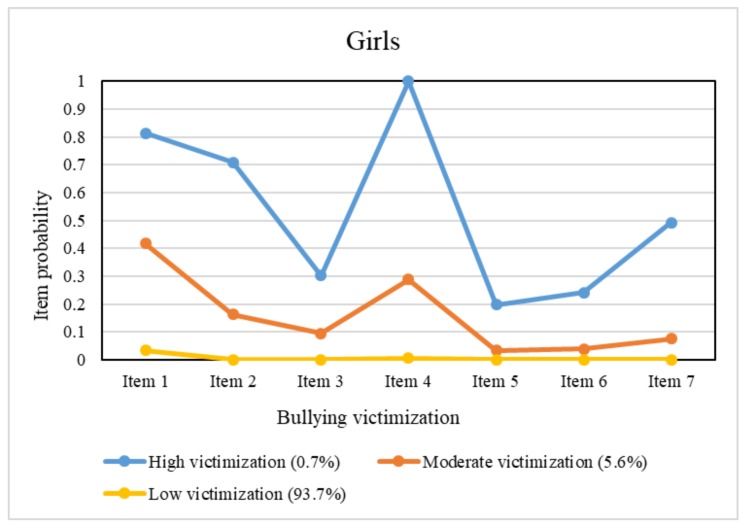
Conditional item probability plots for girls.

The conditional probability plots are shown in Figure 1 for boys and Figure 2 for girls. Discrepant patterns of bullying victimization emerged by gender. For both boys and girls, a majority of the samples (84.2% of boys and 93.7% of girls) had extremely low probabilities or even zero probability for any of the victimization items and were identified as belonging to the low victimization class. There were three additional classes among boys. A small percentage of boys (0.6%) were in a class that had a high probability of endorsing each victimization item; therefore, this class was identified as the high victimization class. The next class comprised 2.8% of boys and was referred to as the moderate victimization class since it exhibited a moderate probability falling between the low victimization class and the high victimization class on each item. The final class, comprising 12.4% of boys, was characterized by the highest probability for a verbal victimization item (item 1) and minimal probabilities for the other items; thus, we referred to this class as the verbal victimization class.

Unlike boys, there were only two additional classes among girls beyond the low victimization class. The smallest class contained 0.7% of girls and was referred to as the high victimization class, which had a relatively high probability for each item, in particular for verbal and relational victimization items (items 1, 2, and 4). The remaining class (5.6%), which had a similar contour of the probability plot as that of the high victimization class of girls, was characterized by a moderate probability for each item compared with that of the high victimization class and the low victimization class; thus, we referred to this class as the moderate victimization class.

### 3.3. Class Membership and Mental Health Outcomes

Equality tests of the means or probabilities across the victimization classes were conducted to examine the associations between bullying victimization class membership and mental health outcomes, including anxiety symptoms, depressive symptoms, NSSI, suicidal ideation, and suicide attempts (Table 3). For both genders, the mean GAD-7 and CES-D scores and probabilities of NSSI, suicidal ideation, and suicide attempts were significantly different (*p* < 0.05) across all classes, with the exception of the comparison of suicidal ideation between the high victimization class and the moderate victimization class among boys. These results of the pairwise comparisons indicated a general trend for both genders that students with higher involvement in bullying victimization had more severe anxiety symptoms and depressive symptoms as well as a higher probability of engaging in NSSI and considering and attempting suicide.

## 4. Discussion

In the present study, we employed an LCA approach to explore patterns of bullying victimization in a large sample of Chinese adolescents. Notably, we found that distinct patterns of victimization existed between boys and girls, with different numbers and characteristics of latent classes by gender in the LCA models. Moreover, class differences were also found in terms of students’ anxiety symptoms, depressive symptoms, NSSI, suicidal ideation and suicide attempts. This result demonstrated the association of bullying victimization with mental health problems.

As expected, most of the students (84.2% of boys and 93.7% of girls) had minimal probabilities for each victimization item and were classified into the low victimization class. The proportion of this reference class was higher than that reported in American studies [21,22]; in other words, Chinese students may generally have a relatively low probability of being bullied. One possible explanation is that the cultural characteristics and social environment of China have long been influenced by Confucian culture. The values of benevolence, righteousness, and propriety advocated by Confucian culture require people to love, help and respect each other, which establishes basic moral principles for the society and plays a role in restraining the bullying behaviors of Chinese adolescents. [37]. On the other hand, the proportion of boys in the low victimization class was lower than that of girls, which is a similar finding to previous studies that bullying victimization was more common among boys [38,39,40].

For the remaining students, characteristics of the item probabilities in the latent classes differed between boys and girls. There was a small number of students (0.56% of boys and 0.72% of girls) who were classified into the high victimization class, wherein boys had a high probability greater than 0.70 for each item. For girls in this class, the item probabilities were found to be heterogenous; they had high probabilities greater than 0.70 for items 1, 2, and 4, representing verbal and relational victimization and had a moderate probability approaching 0.50 for item 7, representing cyber victimization, whereas the probabilities for the other items were relatively low, ranging from 0.20 to 0.30. The moderate victimization class accounted for 2.8% of boys and 5.6% of girls and showed a similar contour of the probability plot to that of the high victimization class of the same gender. There was an extra group among the boys that we identified as the verbal victimization class. It comprised 12.4% of boys, all of whom reported “being called mean names, made fun of, or teased in a hurtful way” but had minimal probabilities for the other items.

Based on the classification and item probabilities, patterns of bullying victimization among students can be summarized. For those at moderate to high risk of bullying victimization, boys were vulnerable to all types of bullying behaviors, while girls were more likely to be victimized by verbal and relational bullying than physical bullying. Moreover, verbal victimization was likely to occur alone in a certain group of boys, but this phenomenon was not observed in girls. These findings are not completely consistent with those of previous studies. For example, research among adolescents in ten Europe countries reported that boys were more likely to be physically and verbally bullied, whereas girls were more prone to be victims of relational bullying [41]. Similar results were found in American and Dutch adolescents [42,43]. A study from South Korea noted that boys were more likely to experience physical bullying, while girls were more likely to experience relational, verbal and cyber bullying [44]. The differences in conclusions may arise from the different sample sources and statistical analysis methods. Similar to our study, Wang et al. used an LCA approach to examine the co-occurrence of victimization subtypes among US adolescents [21]. Specifically, they classified samples into three latent classes labeled “all-types victims”, “verbal/relational victims”, and “non-victims”. However, their study showed that the overall pattern of victimization was the same across genders, which was inconsistent with our finding. Another study [45] of peer victimization conducted an LCA and identified a four-class pattern for middle school students and a three-class pattern for high school students. With regard to gender differences, boys were more likely to be in the “verbal and physical victimization” class, while girls were more likely to be in the “verbal and relational victimization” class. According to the LCA findings in Nylund’s study [22], victims were likely to experience multiple types of bullying rather than a single type. Nevertheless, some boys in our study were only verbal victims without experiencing any other type of victimization. In sum, discrepancies in these LCA results to some extent reveal the diversity and complexity of bullying victimization patterns among adolescents.

Mean levels of anxiety symptoms and depressive symptoms and probabilities of NSSI, suicidal ideation, and suicide attempts were compared across the latent classes. We found that, in general, students with higher involvement in bullying victimization had poorer mental health. This finding is consistent with previous studies demonstrating that bullying victimization is positively associated with mental health problems such as anxiety, depression, NSSI, suicidal ideation, and suicide attempts [3,5,18,46]. Importantly, boys in the verbal victimization class reported more severe problems than those in the low victimization class, signifying that even a single type of victimization could adversely affect victims’ mental health. In addition to the negative consequences included in our study, bullying victimization may also lead to psychosomatic complaints, difficulty sleeping, low self-esteem, poor academic achievement, and poor psychosocial adjustment [47]. Therefore, zero-tolerance policies should be adopted for the prevention of and intervention in peer bullying.

To our knowledge, this is the first study to use LCA to examine patterns of bullying victimization in a large sample of Chinese adolescents and to find distinct patterns between genders. However, several limitations should be noted. First, our sample was limited to Guangdong Province in China. Thus, the findings may not be generalized to the whole country. Second, due to the cross-sectional nature of the study, it is difficult to draw causal inferences based on the associations between class membership and mental health outcomes. Third, as the data were collected based on students’ self-reports, we could not completely avoid recall bias and reporting bias. Finally, covariates that may predict individuals’ bullying victimization class memberships were not included in the LCA models.

## 5. Conclusions

The current study provides a basis for further understanding bullying victimization patterns among Chinese adolescents. Through LCA, the victimization patterns of boys and girls could be best represented by a four-class model and a three-class model, respectively, and there were gender differences in the experiences of various types of bullying victimization. Furthermore, significant differences in mental health outcomes were found across the latent classes, suggesting that bullying victimization is correlated with students’ mental health problems. Consequently, comprehensive interventions in the aspects of legislation, institution setting, parent training, school management and moral education should be implemented to prevent and reduce the occurrence of any type of bullying victimization and the negative effects of bullying victimization on mental health.

## Figures and Tables

**Table 1:** Descriptive statistics of the bullying victimization items and mental health outcomes.

Variable	Value	Total, *n* (%)	Boys, *n* (%)	Girls, *n* (%)	*χ*2/*t* *	*p*-Value
Bullying victimization #						
Item 1	0	18,407 (88.8)	8858 (85.1)	9549 (92.6)	297.09	<0.001
	1	2315 (11.2)	1554 (14.9)	761 (7.4)		
Item 2	0	20,182 (97.4)	10,095 (97.0)	10,087 (97.8)	15.86	<0.001
	1	540 (2.7)	317 (3.0)	223 (2.2)		
Item 3	0	20,169 (97.3)	9982 (95.9)	10,187 (98.8)	172.02	<0.001
	1	553 (2.7)	430 (4.1)	123 (1.2)		
Item 4	0	19,836 (95.7)	9930 (95.4)	9906 (96.1)	6.39	0.012
	1	886 (4.3)	482 (4.6)	404 (3.9)		
Item 5	0	20,488 (98.9)	10,232 (98.3)	10,256 (99.5)	67.37	<0.001
	1	234 (1.1)	180 (1.7)	54 (0.5)		
Item 6	0	20,487 (98.9)	10,234 (98.3)	10,253 (99.4)	61.82	<0.001
	1	235 (1.1)	178 (1.7)	57 (0.6)		
Item 7	0	20,372 (98.3)	10,182 (97.8)	10,190 (98.8)	34.07	<0.001
	1	350 (1.7)	230 (2.2)	120 (1.2)		
GAD-7 scores (M, SD)		4.15 (4.4)	3.57 (4.3)	4.73 (4.5)	19.06	<0.001
CES-D scores (M, SD)		14.36 (10.2)	12.95 (9.4)	15.78 (10.7)	20.21	<0.001
NSSI	No	17,842 (86.1)	9225 (88.6)	8617 (83.6)	109.12	<0.001
	Yes	2880 (13.9)	1187 (11.4)	1693 (16.4)		
Suicidal ideation	No	17,037 (82.2)	9096 (87.4)	7941 (77.0)	378.71	<0.001
	Yes	3685 (17.8)	1316 (12.6)	2369 (23.0)		
Suicide attempts	No	19,968 (96.4)	10,170 (97.7)	9798 (95.0)	103.12	<0.001
	Yes	754 (3.6)	242 (2.3)	512 (5.0)		

# For the bullying victimization items, frequencies of two or three times a month or more were coded as 1, and others were coded as 0; * the *χ*^2^ test was used for the bullying victimization items, NSSI, suicidal ideation and suicide attempts; the *t* test was used for the GAD-7 and CES-D scores; NSSI: non-suicidal self-injury.

**Table 2:** Fit indices for the latent class models.

Classes	AIC	BIC	SSABIC	Entropy	LMR (*p*)	BLRT (*p*)
	**Boys (*n* = 10,412)**					
2	21,207.705	21,316.465	21,268.798	0.911	<0.001	<0.001
3	20,893.680	21,060.446	20,987.355	0.881	<0.001	<0.001
4	20,858.694	21,083.466	20,984.953	0.961	<0.001	<0.001
5	20,855.133	21,137.911	21,013.974	0.845	0.156	0.050
	**Girls (*n* = 10,310)**					
2	12,976.340	13,084.953	13,037.285	0.926	<0.001	<0.001
3	12,918.702	13,085.242	13,012.151	0.851	0.006	<0.001
4	12,901.066	13,125.533	13,027.019	0.875	0.049	<0.001
5	12,887.763	13,170.157	13,046.220	0.886	0.105	<0.001

AIC: Akaike information criterion; BIC: Bayesian information criterion; SSABIC: sample size adjusted Bayesian information criterion; LMR: Lo-Mendell-Rubin adjusted likelihood ratio test; BLRT: bootstrapped likelihood ratio test.

**Table 3:** Comparisons of mental health outcomes across the latent classes.

**Boys**	**High Victimization**	**Moderate Victimization**	**Verbal Victimization**	**Low Victimization**
GAD-7 scores	12.59 (1.09) ^a^	7.47 (0.41) ^b^	4.55 (0.15) ^c^	3.19 (0.04) ^d^
CES-D scores	33.22 (2.11) ^a^	23.90 (0.81) ^b^	15.75 (0.31) ^c^	11.93 (0.09) ^d^
NSSI	0.54 (0.08) ^a^	0.33 (0.03) ^b^	0.18 (0.05) ^c^	0.09 (0.01) ^d^
Suicidal ideation	0.41 (0.08) ^a^	0.43 (0.03) ^a^	0.16 (0.02) ^b^	0.10 (0.01) ^c^
Suicide attempts	0.27 (0.07) ^a^	0.10 (0.02) ^b^	0.03 (0.01) ^c^	0.02 (0.01) ^d^
**Girls**	**High Victimization**	**Moderate Victimization**	**Low Victimization**	
GAD-7 scores	11.69 (0.89) ^a^	9.05 (0.33) ^b^	4.26 (0.05) ^c^	
CES-D scores	35.18 (1.64) ^a^	27.80 (0.73) ^b^	14.46 (0.12) ^c^	
NSSI	0.62 (0.06) ^a^	0.42 (0.03) ^b^	0.13 (0.01) ^c^	
Suicidal ideation	0.67 (0.06) ^a^	0.52 (0.03) ^b^	0.19 (0.01) ^c^	
Suicide attempts	0.35 (0.07) ^a^	0.19 (0.03) ^b^	0.04 (0.01) ^c^	

The means and standard errors were calculated for the GAD-7 and CES-D scores; the probabilities and standard errors were calculated for NSSI, suicidal ideation and suicide attempts; different letters in superscript indicate statistical significance at an α of 0.05; NSSI: non-suicidal self-injury.
